# A ruptured dissecting aneurysm of the anterior radiculomedullary artery caused by hypoplastic vertebral artery angiography: case report

**DOI:** 10.1186/s12883-025-04517-6

**Published:** 2025-12-12

**Authors:** Wenwen Chen, Xiu Shen, Yindan Yao

**Affiliations:** 1 Department of Internal medicine, Yuyao Hospital of Traditional Chinese Medicine, Yuyao, Zhejiang China; 2 Department of Hematology and Oncology, Ningbo No.2 Hospital, Ningbo, Zhejiang China; 3 Department of Neurology, Ningbo No.2 Hospital, Ningbo, Zhejiang China

**Keywords:** Hypoplastic vertebral artery, Angiography, Anterior radiculomedullary artery, Dissecting aneurysm, Case report

## Abstract

**Background:**

Cerebral digital subtraction angiography (DSA) is increasingly utilized in the diagnosis of cerebrovascular disorders. We report a rare case of a ruptured dissecting aneurysm involving the anterior radiculomedullary artery of the cervical spinal cord, precipitated by angiography of a hypoplastic vertebral artery.

**Case presentation:**

Cerebral computed tomography angiography (CTA) revealed severe stenosis of the M1 segment of the right middle cerebral artery in a 70-year-old woman who presented with paroxysmal weakness affecting the left upper and lower limbs. Dual antiplatelet therapy with aspirin and clopidogrel was initiated. On day 3, cerebral digital subtraction angiography (DSA) was performed. During continuous contrast injection into the right hypoplastic vertebral artery, the anterior radiculomedullary artery—a branch of the vertebral artery at the C6 vertebral level—underwent luminal dilation and subsequently ruptured, resulting in extensive extravasation of contrast medium into the spinal subarachnoid space. Non-contrast head CT confirmed widespread subarachnoid hemorrhage. Following 10 days of conservative medical management, follow-up head CT demonstrated complete resolution of the hemorrhage. The patient was discharged without significant neurological deficits and remained free of recurrent subarachnoid hemorrhage during a 3-year clinical follow-up period.

**Conclusions:**

The occurrence of a ruptured dissecting aneurysm of the anterior radiculomedullary artery induced by vertebral angiography is exceedingly rare. We hypothesize that the sustained high-pressure and high-volume injection of contrast medium was responsible for the arterial dissection and subsequent rupture. During interventional procedures, neurointerventional radiologists should avoid selective angiography of small, hypoplastic vertebral arteries whenever possible to minimize the risk of such complications.

**Supplementary Information:**

The online version contains supplementary material available at 10.1186/s12883-025-04517-6.

## Background

Isolated aneurysms of the anterior radiculomedullary artery in the cervical spinal cord are exceedingly rare. Hemodynamic abnormalities have been recognized as significant contributing factors in the formation of such aneurysms, particularly given that a substantial proportion are associated with underlying cerebrovascular pathologies [1–4]. Cerebral digital subtraction angiography (DSA) is increasingly employed in the diagnostic evaluation of cerebrovascular disorders. In this report, we describe a rare case of a ruptured dissecting aneurysm of the anterior radiculomedullary artery at the cervical level, precipitated by angiography of a hypoplastic vertebral artery. We hypothesize that sustained high-pressure and high-volume injection of contrast medium led to mechanical stress on the vessel wall, resulting in arterial dissection and subsequent rupture.

## Case presentation

A 70-year-old female was admitted to Ningbo No. 2 Hospital on May 31, 2021, presenting with a three-day history of paroxysmal weakness in the left upper and lower limbs. Her medical history included hypertension of five years’ duration. Neurological examination upon admission was unremarkable, with no evidence of sensory deficits, motor impairment, or sphincter dysfunction. Cerebral computed tomography angiography (CTA) performed post-admission revealed severe stenosis of the M1 segment of the right middle cerebral artery. Dual antiplatelet therapy with aspirin and clopidogrel, along with standard secondary prevention measures, was initiated.

**Fig. 1:**
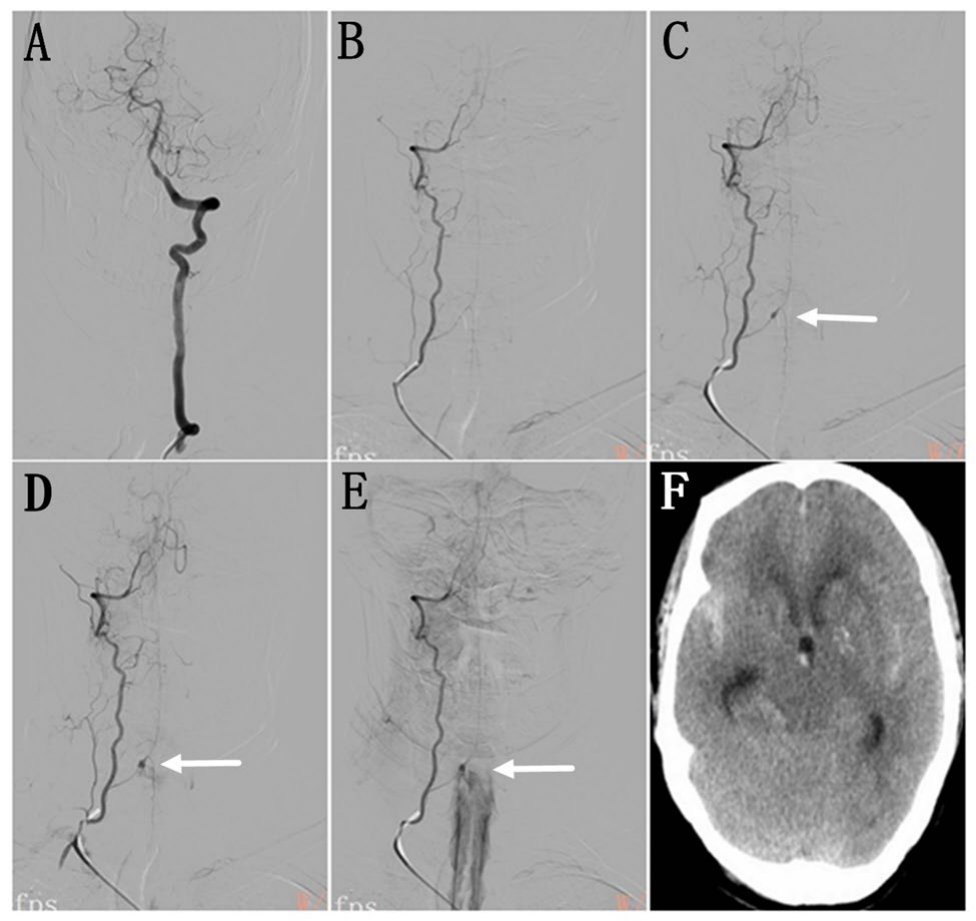
Bilateral vertebral artery angiography and Computed tomography (CT). **A** Left vertebral angiography showed the normal dominant vertebral artery. **B** Right vertebral angiography showed the hypoplastic vertebral artery and normal filling of the anterior radiculomedullary artery in the early arterial phase. **C**-**E** The subsequent angiography showed the formation and rupture of a fusiform dissecting aneurysm (arrows) in the intradural segment of the anterior radiculomedullary artery. **F** Head CT showed extensive subarachnoid hemorrhage

On day 3 of hospitalization, digital subtraction angiography (DSA) was performed via femoral artery access using a 5-F VER catheter (Cordis, USA) for further evaluation of the cerebral vasculature. At our institution, iodixanol (Hengrui Medical, China) is the contrast agent of choice for cerebral angiography. Standard injection parameters were set as follows: for internal carotid artery angiography, 4 mL/s at a pressure limit of 300 pounds per square (PSI), total volume 7 mL; for vertebral artery angiography, 3 mL/s at 300 PSI, total volume 5 mL. Using these settings, selective angiography of the bilateral internal carotid arteries and the left vertebral artery was successfully completed (Fig. 1A). Due to the small caliber of the right vertebral artery, injection parameters were adjusted to a rate of 2.5 mL/s and a volume of 4 mL; however, the pressure limit remained unchanged at 300 PSI. During continuous contrast injection, the anterior radiculomedullary artery—a branch of the vertebral artery at the C6 vertebral level—underwent luminal dilation followed by rupture, resulting in extensive extravasation of contrast medium into the spinal subarachnoid space (Fig. 1B–E and Video 1, Supplementary Material). The patient immediately developed severe headache and agitation. After 15 min of observation, repeat angiography of the right subclavian artery demonstrated marked improvement with cessation of contrast leakage from the rupture site. Vital signs remained stable, and neurological assessment revealed alertness, neck stiffness, and preserved limb strength. The patient was subsequently transferred to the intensive care unit for close monitoring. Antiplatelet therapy was discontinued. Given the presence of symptomatic intracranial atherosclerotic stenosis, prohemostatic agents were avoided. On post-procedure day 2, non-contrast head CT confirmed extensive subarachnoid hemorrhage (Fig. 1F). Following 10 days of conservative management, follow-up head CT showed complete resolution of the hemorrhage. The patient was discharged without significant neurological deficits. At 3-year clinical follow-up via telephone interview, the patient remained free of recurrent subarachnoid hemorrhage. However, she declined further imaging evaluations, including CTA or DSA.

## Discussion

A review of the literature indicates that anterior radiculomedullary artery aneurysm in the cervical spinal cord as a complication of vertebral angiography is rare but not unprecedented. Taniuria and colleagues reported a similar adverse event associated with vertebral angiography [5]. The authors suggested that during contrast medium injection, the catheter tip was lodged within a branch of the vertebral artery at the level of the C6 vertebral body, and subsequent high-pressure, high-volume contrast injection led to dissection of the anterior radiculomedullary artery.

The present case exhibits several distinguishing features compared to the previously reported case. In our patient, the right vertebral artery measured approximately 2 mm in diameter, consistent with a hypoplastic vessel. Performing selective angiography in such a small-caliber artery may have been an avoidable intervention. During the early arterial phase, the right vertebral artery and its branches were adequately opacified without evidence of an aneurysm. However, in the late arterial phase, sudden focal dilation of the anterior radiculomedullary artery at the C6 level was observed, rapidly progressing to rupture with extravasation of contrast medium into the spinal subarachnoid space. This temporal evolution suggests that the artery was initially intact, and mechanical injury due to sustained high-pressure and high-volume contrast injection likely precipitated dissection and rupture.

This case carries important clinical and educational implications. First, selective angiography of small, hypoplastic vertebral arteries using continuous high-pressure and high-volume contrast injection poses significant risk. It is strongly recommended that injection rates and volumes be minimized during selective vertebral angiography. This principle should also be applied to the catheterization of other small-diameter arteries. Second, when dealing with a hypoplastic vertebral artery, selective angiography should be avoided unless clinically necessary. Ipsilateral subclavian artery angiography may serve as a safer alternative for assessing downstream flow. Furthermore, if selective catheterization is required, the use of a smaller-diameter catheter—such as a 4-F or even a microcatheter—may reduce hemodynamic stress on fragile vessels and enhance procedural safety.

## Conclusions

In conclusion, the occurrence of a ruptured dissecting aneurysm of the anterior radiculomedullary artery following vertebral angiography is exceedingly rare. We hypothesize that sustained high-pressure and high-volume injection of contrast medium was responsible for the arterial dissection and subsequent rupture. During interventional procedures, neurointerventional radiologists should avoid selective angiography of small, hypoplastic vertebral arteries whenever possible to minimize the risk of such complications.

## Supplementary Information

Supplementary Material 1.

Supplementary Material 2.

## Data Availability

Please contact the authors for data requests.

## Abbreviations

DSA: Digital subtraction angiography.
CT: Computed tomography.
CTA: Computed tomography angiography.
PSI: Pounds per square.
