# Supplementary material for: A ruptured dissecting aneurysm of the anterior radiculomedullary artery caused by hypoplastic vertebral artery angiography: case report
Source: BMC Neurol. 2025 Dec 12;25:500. doi: 10.1186/s12883-025-04517-6 (PMC12699801; doi:10.1186/s12883-025-04517-6)
Supplement: Supplementary file 2 — Supplementary Material 2. [file 12883_2025_4517_MOESM2_ESM.docx]

Video 1. Right hypoplastic vertebral angiography showed the formation and rupture process of the anterior radiculomedullary artery dissecting aneurysm.
